# Investigation of single-domain Au silicide nanowires on Si(110) formed for Au coverages in the monolayer regime

**DOI:** 10.1038/s41598-021-94106-7

**Published:** 2021-07-20

**Authors:** Stephan Appelfeller

**Affiliations:** MAX IV Laboratory, Lund University, 221 00 Lund, Sweden

**Keywords:** Nanowires, Surfaces, interfaces and thin films

## Abstract

The self-organized formation of single domain Au silicide nanowires is observed on Si(110). These nanowires are analysed using scanning tunnelling microscopy (STM) and spectroscopy (STS) as well as photoemission spectroscopy (PES). Core-level PES is utilised to confirm the formation of Au silicide and establish its presence as the top most surface structure, i.e., the nanowires. The growth of the Au silicide nanowires and their dimensions are studied by STM. They form for Au coverages of about 1 monolayer and are characterized by widths of about 2 to 3 nm and heights below 1 nm while reaching lengths exceeding 500 nm when choosing appropriate annealing temperatures. Valence band PES and STS indicate a small but finite density of states at the Fermi level typical for compound metals.

## Introduction

The Au–Si material system has drawn interest since decades, but nevertheless showed new enticing results in recent years. The Au–Si phase diagram is of simple eutectic nature without any stable solid Au silicide^1^. This property is utilised for the growth of pure Si nanowires in vapour–liquid–solid epitaxy, in which Au droplets are used as nucleation centres^2^. It is of course possible to achieve the formation of metastable Au silicides using highly non-equilibrium methods, e.g., quenching of liquid Au–Si alloys from high temperatures^3–9^. Intriguingly at any Au–Si interface, Si may diffuse into Au leading to the formation of a stable interface Au silicide even at room temperature^10–13^. Furthermore, the presence of Au silicides on top of thick Au films deposited on clean Si substrates was reported^14–17^. Thus, while there is no stable bulk Au silicide, it may form in lower dimension, e.g., at two-dimensional surfaces or interfaces.

For low Au coverages on Si substrates, many enticing Au induced surface reconstructions have been investigated, e.g., the $5 \times 2$ reconstruction on Si(111) showing interesting self doping effects by Si adatoms^18,19^, atomic Au chains on vicinal Si(111) substrates having quasi-one-dimensional properties and hosting spin chains^20–25^, or the $2 \times 5$ reconstruction on Si(110) characterised by a quasi-one-dimensional electronic structure^26,27^. Furthermore, the growth of Au silicide nanostructures was reported for higher Au coverages on various Si substrates^28–32^.

In this report, we study the formation of Au silicide nanowires on Si(110) substrates. In addition to the above mentioned $2 \times 5$ reconstruction, there are an Au induced $1 \times 2$ reconstruction for very low Au coverages and a $\left( 4 , 0\right) \times \left( {\overline{1}} , 3\right)$ superstructure for higher Au coverages than needed for the $2 \times 5$ reconstruction^26,33,34^. By further increasing the Au coverage to about 1 monolayer (ML), a streak structure dominates low energy electron diffraction (LEED) images. The surface structure leading to these streaks are the here studied Au silicide nanowires.

In addition to LEED, the Au silicide nanowires were investigated using scanning tunnelling microscopy (STM) and spectroscopy (STS) as well as photoemission spectroscopy (PES). The core-level PES spectra show surface related components with core-level shifts typical for Au and Si in vicinity of each other proving that the nanowires are made out of an Au–Si compound. The narrow nanowires with widths of about only 2 to $3~{\mathrm {nm}}$ form single domain arrays and have high aspect ratios only limited by the substrate miscut when high enough annealing temperatures up to $750\,^\circ {\mathrm {C}}$ are utilised during their preparation. They show a small but finite density of states at the Fermi level typical for metallic compounds and are stable for days at room temperature.

## Results and discussion

The basis of any study on surface nanostructures is the use of well-defined substrates. Thus, the Si(110) substrate is briefly described before the Au silicide nanowires are introduced and some of their properties discussed in more detail subsequently.

### The clean Si(110) substrate

In this work, clean Si(110) substrates were used as template for the growth of Au silicide nanowires. The clean Si(110) surface is characterised by the so-called $16 \times 2$ reconstruction^35^. This reconstruction is very sensitive to contaminations, e.g., as little as $0.007~{\mathrm {ML}}$ Ni lead to a $5 \times 1$ reconstruction^36^. The successful preparations of clean substrates was always controlled by LEED. Additionally, STM and PES measurements were routinely used to confirm the LEED results in the STM and PES ultra-high vacuum (UHV) chamber systems, respectively.

**Figure 1:**
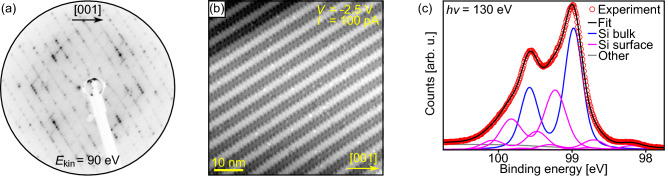
(**a**) LEED image, (**b**) STM image, and (**c**) Si *2p* spectrum of clean Si(110) substrates.

A typical LEED image of a clean substrate is shown in Fig. 1a. The observation of densely dotted lines along the $\left[ 1{\overline{1}}1\right]$ and $\left[ 1{\overline{1}}{\overline{1}}\right]$ directions agrees very well with earlier studies^35,37,38^. In Fig. 1a, the spots of one of the two domains have a higher intensity indicating the dominant formation of one domain. In STM, the detailed appearance of the so-called $16 \times 2$ reconstruction is strongly dependent on the used tunnelling parameters, but stripes along the $\left[ 1{\overline{1}}{\overline{2}}\right]$ and $\left[ 1{\overline{1}}2\right]$ directions are always observed at larger scales^36,39–42^. For the sample with the LEED image in Fig. 1a, an STM image is depicted in Fig. 1b. As expected from the LEED data, the dominant formation of one domain is also found in STM. The STM image also reveals a low defect density on the atomic scale, which is only possible for clean substrates with negligible contaminations.

Using PES, the cleanness of a substrate may be judged in a first step by survey spectra covering a wide binding energy range. If only Si related lines are observed the Si substrate is presumably contamination free. In a second step, high-resolution Si *2p* spectra may be analysed in more detail. As shown in Fig. 1c, the Si *2p* line of a clean Si(110) surface can be described using six spin-orbit-split components, one originating from the Si bulk and five related to surface Si sites. The here found spectral composition with surface-core-level shifts of $-0.79~{\mathrm {eV}}$, $-0.29~{\mathrm {eV}}$, $0.25~{\mathrm {eV}}$, $0.50~{\mathrm {eV}}$, and $0.70~{\mathrm {eV}}$ with respect to the binding energy of the bulk component as well as the relative intensities of the spectral components agree very well with literature data^41,43^.

### Au silicide nanowires

There are conflicting reports about the forming surface structure for a Si(110) substrate covered by $1~{\mathrm {ML}}$ Au. Kang and co-workers observed no ordered structure in LEED^26^, while the $\left( 4 , 0\right) \times \left( {\overline{1}} , 3\right)$ reconstruction was observed with reflection high energy electron diffraction (RHEED) for samples at high temperatures by Ino^33,34^. In addition, Yamamato observed streaks in RHEED for such an Au coverage when the sample was cooled down to room temperature after annealing^34^.

**Figure 2:**
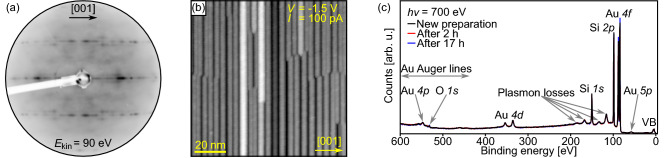
(**a**) LEED image, (**b**) STM image, and (**c**) survey PES spectra of Au silicide nanowire preparations. The nanowires were prepared by depositing about $1.0~{\mathrm {ML}}$ Au on a clean Si(110) substrate at room temperature followed by annealing at $600\,^\circ {\mathrm {C}}$ for $2~{\mathrm {min}}$.

In this work, streaky features are observed in LEED at room temperature after depositing about $1~{\mathrm {ML}}$ Au on a Si(110) substrate and annealing at $600\,^\circ {\mathrm {C}}$ confirming the results from Yamamato (Fig. 2a)^34^. All streaks are oriented along the $\left[ 001 \right]$ direction and show intensity variations indicating the growth of extended structures in $\left[ {\overline{1}}10 \right]$ direction that form an irregular array, e.g., similar as Tb silicide nanowires on vicinal Si(001) substrates^44^. In the LEED image, there are not only streaks along the main axes but also shifted ones. Their separation from the main axes indicates a $\times 10$ periodicity in $\left[ {\overline{1}}10 \right]$ direction. This observation agrees again very well with the RHEED experiments by Yamamato, where also off-axis streaks were reported^34^.

The expectation of extended structures in $\left[ {\overline{1}}10 \right]$ direction is confirmed by STM measurements. Figure 2b shows an STM image of another sample prepared by depositing about $1~{\mathrm {ML}}$ Au on a Si(110) substrate and annealing at $600\,^\circ {\mathrm {C}}$. Narrow and straight nanowires formed. They are characterized by homogeneous widths along their lengths, very large aspect ratios, and only few kinks. However, they do not constitute a well-ordered array since the widths and the heights of the nanowires, as well as the separations between them vary.

In contrast to the so-called $16 \times 2$ reconstruction of the clean Si(110) surface, the nanowires grow only in one single domain. The Si(110) surface is two-fold symmetric and the $\left[ 001\right]$ direction is the mirror symmetry axis. Consequently, the clean surface shows that shows stripes in the $\left[ 1{\overline{1}}2\right]$ direction also shows stripes in the equivalent $\left[ {\overline{1}}12\right]$ direction. The nanowire growth direction, the $\left[ {\overline{1}}10 \right]$ direction, is perpendicular to the mirror symmetry axis, so that its equivalent direction, the $\left[ 1{\overline{1}}0 \right]$ direction, is antiparallel to it leading to single domain growth.

That the observed nanowires are induced by the Au deposition can be confirmed by survey PES spectra as shown in Fig. 2c. Only Si and Au related lines and minuscule intensity from the O *1s* level are observed. Consequently no other substance, e.g., W from the evaporator filament, can be the source for the nanowire formation. In addition, it has to be noted that the photoelectrons with a binding energy of about $535~{\mathrm {eV}}$ may not stem dominantly from the O core level since an Au auger line also contributes at this binding energy for the used photon energy^45^. Thus, the oxidation of the nanowires is even less than expected from the first view of the survey PES spectra.

#### Nanowire formation

**Figure 3:**
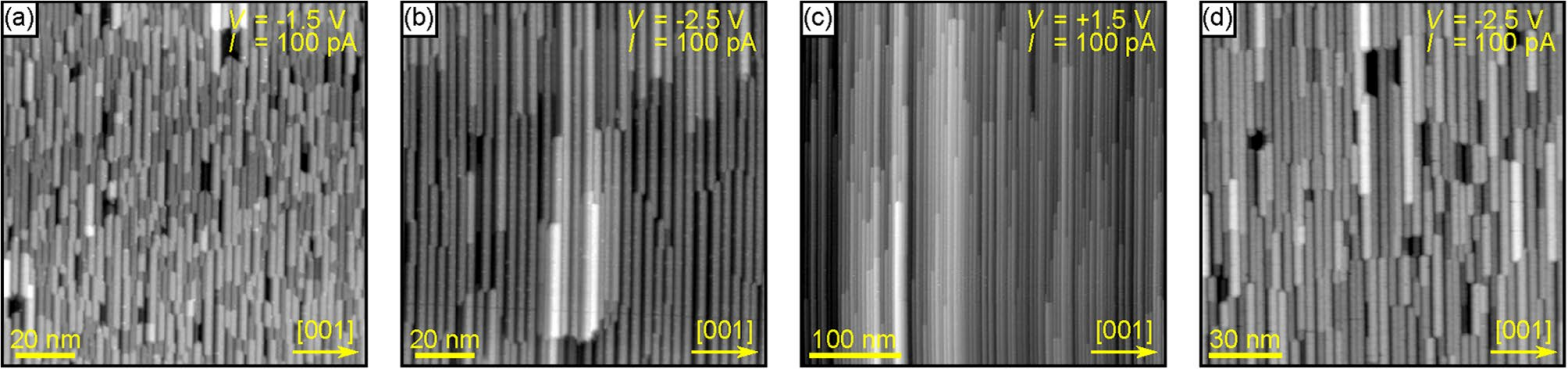
STM images of various Au silicide nanowire preparations. (**a**) About $1.0~{\mathrm {ML}}$ Au deposited at room temperature followed by annealing at $400\,^\circ {\mathrm {C}}$ for $2~{\mathrm {min}}$. (**b**) The sample shown in (**a**) additionally annealed at $600\,^\circ {\mathrm {C}}$ for $2~{\mathrm {min}}$. (**c**) About $1.0~{\mathrm {ML}}$ Au deposited on a sample heated to $500\,^\circ {\mathrm {C}}$. (**d**) About $1.5~{\mathrm {ML}}$ Au deposited at room temperature followed by annealing at $600\,^\circ {\mathrm {C}}$ for $2~{\mathrm {min}}$.

The homogeneity of the forming nanowire ensemble is strongly dependent on the preparation conditions. In Fig. 3, the STM images of four different preparations show clear variations in nanowire growth.

When the nanowires are prepared by deposition of about $1~{\mathrm {ML}}$ Au at room temperature followed by annealing with a low annealing temperature of only $400\,^\circ {\mathrm {C}}$, their growth appears irregular (Fig. 3a). There are many short nanowires leading a very small median length of the nanowires of only about $13~{\mathrm {nm}}$. The nanowires have an increased length with their median length larger than $50~{\mathrm {nm}}$ when the sample is additionally annealed at $600\,^\circ {\mathrm {C}}$ (Fig. 3b). This length increase leads to a more ordered surface appearance. In contrast to the length, the width of the nanowires is only marginally influenced by the additional anneal leading to much higher aspect ratios of the nanowires after the second anneal. For the here shown preparations, the median full width at half maximum of the nanowires increased from 2.0 to $2.2~{\mathrm {nm}}$. This difference is mainly related to the increased separation between nanowires after the second anneal leading to apparently deeper grooves between nanowires and consequently apparently increased widths. By comparing Figs. 2b and 3b, it is evident that a preceding annealing at $400\,^\circ {\mathrm {C}}$ does not influence the surface morphology after annealing at $600\,^\circ {\mathrm {C}}$.

Nanowires with even larger aspect ratios can be observed when the sample is not annealed after the Au deposition, but already during the Au deposition (Fig. 3c). Using a moderate annealing temperature of $500\,^\circ {\mathrm {C}}$, nanowires with lengths exceeding $500~{\mathrm {nm}}$ are found and, in general, the nanowire length is mainly limited by the wafer miscut in the nanowire growth direction. In contrast, the full width at half maximum is only slightly increased to about $3~{\mathrm {nm}}$.

The same Au coverage of about $1.0~{\mathrm {ML}}$ was used for all above discussed preparations. When the coverage is increased to about $1.5~{\mathrm {ML}}$, annealing the sample at $600\,^\circ {\mathrm {C}}$ after the Au deposition is not sufficient any more to enable the dominant formation of nanowires with high aspect ratios (Fig. 3d). However while the nanowire growth is rather irregular, they still show in general the same characteristics, e.g. similar widths below $4~{\mathrm {nm}}$ and a smooth appearance in STM, as the nanowires formed at lower Au coverages. Thus, using even higher annealing temperatures leads again to the formation of nanowires with high aspect ratios, as will be shown in the “Nanowire stability” section.

Summarising this section, nanowires start to form at rather low temperatures for Au coverages of about $1~{\mathrm {ML}}$. Their widths increase slightly with increasing Au coverage and increasing annealing temperature, but this width increase is drastically overcompensate by a length increase when the annealing temperature is high enough leading to nanowires with very high aspect ratios.

#### Metallic behaviour

An important property of any material is its conductivity since there are different application possibilities if the material is a metal, a semiconductor, or an isolator. Semiconductors are characterized by a band gap in their electronic structure while metals have a finite density of states at the Fermi level. Here, the electronic structure is probed locally by STS and globally by PES.

**Figure 4:**
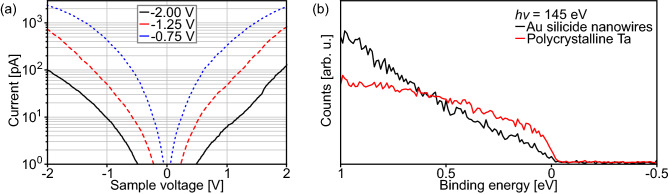
(**a**) STS spectra taken on Au silicide nanowires. Between the spectra the stabilising sample voltage was changed in the way indicated in the figure legend. The stabilising current was always $I=100~{\mathrm {pA}}$. (**b**) PES spectra near the Fermi level for an Au silicide nanowire sample and polycrystalline Ta covered with a small amount of Au as reference.

Figure 4a shows STS spectra taken above a nanowire for varying stabilising conditions of the tip before switching off the feedback loop regulating the tip height and measuring an $I\left( V\right)$-spectrum. Since the stabilising current was the same before every measurement, a reduced absolute value of the sample voltage corresponds to a tip nearer at the nanowire. In the STS spectrum, where the tip was far from the nanowire (black continuous graph in Fig. 4a), there is a wide sample voltage range of about $1~{\mathrm {V}}$, in which negligible current below $1~{\mathrm {pA}}$ is measured. In general, such a behaviour may be related to the existence of a band gap, but, with decreasing tip nanowire distance, this voltage range shrinks to almost nothing indicating that it is only related to the reduced tunnelling current for large tip sample separation. Thus, the nanowires presumably have a finite density of states at the Fermi level.

This result of the STS experiments is confirmed by PES measurements near the Fermi level (binding energy $E_{\mathrm {bin}}=0$) (Fig. 4b). While no clear Fermi edge is resolved, there is photoelectron intensity up to the Fermi level, which was calibrated by a reference measurement on the Ta clamp fixing the Si substrate. It has to be mentioned that no clear Fermi edge is expected for quasi-one-dimensional systems^46^. However, the here found rather low photoelectron intensity near the Fermi level, which is consistent with earlier reports on Au silicide structures formed at room temperature^47^, indicates a similarly low conductivity typical for compound conductors.

#### Structural properties

The observed nanowires are termed Au silicide nanowires throughout the report for an easier understanding of the report, but they may also be pure Au nanowires forming in an island growth mode or pure Si nanowires, e.g., on top of a subsurface Au layer similar to the B induced $\sqrt{3} \times \sqrt{3}$ reconstruction on Si(111)^48,49^ or formed by solid-liquid-solid epitaxy^50,51^. Thus, the nanowire structure has to be discussed more in depth.

The STM images revealed straight nanowires with low variances in their widths both along a single nanowire and of the whole nanowire assembly, which indicates a structural relation between all nanowires. This means that there are at least reoccurring structure motives with certain extensions and orientations leading to structurally similar nanowires and no amorphous structures. In contrast, the nanowire surfaces never showed a reoccurring superstructure in STM, which is consistent with the missing of sharp spots in the LEED images. Thus, one may assume that the nanowire surface structure is determined locally, e.g., by small composition variations, but there is an energetically favourable interface structure between nanowires and substrate leading to the similar nanowire dimensions.

The height of a nanowire, measured from lowest point between nanowires to highest point on top of a nanowire, is typical about $0.4~{\mathrm {nm}}$ and was always lower than $1~{\mathrm {nm}}$. Such heights indicate multilayer nanowires. However, the height difference between apparently different layers of nanowires is $(0.19\pm 0.02)~{\mathrm {nm}}$ coinciding with the nominal Si(110) step height of $0.192~{\mathrm {nm}}$. Thus, substrate steps may be responsible for apparently additional nanowire layers. Furthermore, the disordered appearance of nanowire preparations using low annealing temperatures, e.g., see Fig. 3a, indicates lateral Si transport during nanowire formation since the nanowires do not follow the morphology of the bare substrate with large terraces (Fig. 1b). Consequently, the stoichiometric composition of the nanowires cannot be estimated by relating the nanowire surface coverage to the deposited Au amount. Thus, the insights on the atomic structure of the nanowires gained by STM are limited.

**Figure 5:**
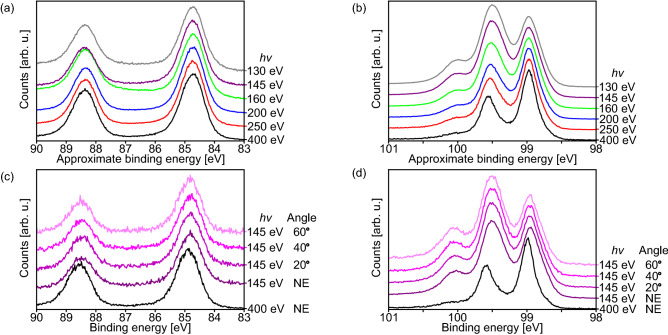
(**a**) Au *4f* and (**b**) Si *2p* spectra of an Au silicide nanowires sample at varying photon energies in normal emission (NE). (**c**) Au *4f* and (**d**) Si *2p* spectra of another Au silicide nanowires sample at varying photon energies and emission angles. Both samples were prepared by depositing about $1.0~{\mathrm {ML}}$ of Au at room temperature and annealing at $600\,^\circ {\mathrm {C}}$. A constant background was subtracted from all spectra, which were then normalized to show the same height for the first distinguishable peak at lowest binding energy. The binding energy scales in (**c**) and (**d**) were calibrated by determination of the Fermi level, which was not possible for (**a**) and (**b**) due to charging effects.

Further information on the nanowire structure is obtainable by analysing PES spectra with varying surface sensitivity. Figure 5a,b show Au *4f* and Si *2p* spectra, respectively, of a nanowire sample where the surface sensitivity was varied by the used photon energy. For the chosen photon energy range, the surface sensitivity is increased for decreased photon energy. Furthermore, Fig. 5c,d depict Au *4f* and Si *2p* spectra, respectively, for another nanowire sample. There the surface sensitivity was not only increased by reducing the photon energy, but also by detuning the photoemission angle away from normal emission.

The agreement between the measurements of the two different samples is very good showing the good reproducibility of the data. The Au *4f* spectra change very little with varying surface sensitivity. Beside the changes of the background intensity, there is a small reduction of the branching ratio of the spin-split components with decreasing photon energy. This is related to the stronger influence of the kinetic energy difference of about $3.7~{\mathrm {eV}}$ on the electron inelastic mean free path for overall lower kinetic energies of the photoelectrons. The Si *2p* spectra change more drastically. With increasing surface sensitivity, the higher binding energy peak rises and shifts slightly. Furthermore, the lower binding energy peak becomes wider with increasing surface sensitivity although the resolution of the setup increases with decreasing photon energy indicating the overlap of multiple components in this peak. This assumption is further confirmed by a small shift of the peak (Fig. 5d).

**Figure 6:**
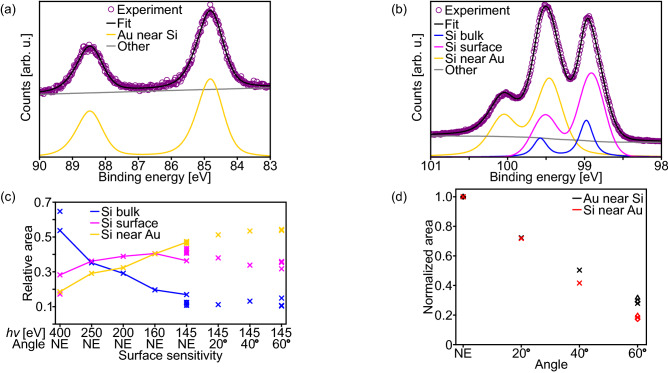
(**a**, **b**) Fitting results of as measured (**a**) Au *4f* and (**b**) Si *2p* spectra of an Au silicide nanowires sample taken with $h\nu =145~{\mathrm {eV}}$ photons at NE. (**c**) Diagram illustrating the relative intensities of the Si *2p* spectral components with respect to the total intensity of the line depending on the surface sensitivity for multiple samples. The lines connect the points obtained by fitting the spectra from Fig. 5b. The circles indicate fits with an asymmetric Au silicide component. If there appear to be fewer data points for a component than for the others at a given surface sensitivity, there are overlapping data points. (**d**) Diagram depicting the area of the Au silicide Si *2p* spectral component and Au silicide Au *4f* line depending on the emission angle and normalized to the area of NE for multiple samples indicated by different symbols.

For a more in-depth analysis, the spectra were least-square fitted. The Au *4f* peak can be very well approximated with a single rather wide component if one allows for small anisotropy parameters (Fig. 6a). The average binding energy of the $4f_{7/2}$ part is $(84.85\pm 0.03)~{\mathrm {eV}}$. This large shift from the position of metallic Au ($84.00~{\mathrm {eV}}$) indicates a strong interaction between the Au overlay and the Si substrate. This shift is in agreement with earlier studies on annealed Au silicide structures on Si(111) substrates^17,52,53^ and is considerably larger than reported for Au silicide layers obtained at room temperature^16,47,54^. The branching ratio reduces from about 0.72 for $h\nu =400~{\mathrm {eV}}$ photons to about 0.58 for $h\nu =145~{\mathrm {eV}}$, while the spin-orbit splitting is $(3,66\pm 0.02)~{\mathrm {eV}}$ taking into account all measurements. The Au *4f* spectra can also be well approximated by two slightly narrower components without any anisotropy. However even using such an approach, there is never a metallic Au component indicating that all of the Au atoms have reacted with Si. Consequently, the here found nanowires cannot be pure Au nanowires.

The Si *2p* spectra can be very well described using three components (Fig. 6b), one narrow component (${\mathrm {B}}$) at a $2p_{3/2}$ binding energy of $(98.99\pm 0.01)~{\mathrm {eV}}$ and two wider components ${\mathrm {Si}}_{\text {S}}$ and ${\mathrm {Si}}_{\text {Au}}$ at $(98.92\pm 0.01)~{\mathrm {eV}}$ and $(99.46\pm 0.01)~{\mathrm {eV}}$, respectively, with the given uncertainties representing the allowed binding energy ranges during the fitting of spectra with calibrated binding energy scale. Typically, bulk components are narrower than surface components since surfaces are not as well ordered as the bulk. Thus, the ${\mathrm {B}}$-component is assigned to the Si bulk. Based on the previous reports on Au silicide structures^16,17,47,52–54^, the highest binding energy component ${\mathrm {Si}}_{\text {Au}}$ can be assigned to Si in Au silicide although the here found chemical shift of this component is slightly lower than the previously reported values. The ${\mathrm {Si}}_{\text {S}}$-component is related to Si at or near the surface, but not bound to Au atoms.

This assignment is in agreement with the observed changes of relative intensities with surface sensitivity (Fig. 6c). The Si bulk component drastically decreases in relative intensity when the surface sensitivity is increased by decreasing the photon energy from 400 to $145~{\mathrm {eV}}$ and remains low when the surface intensity is further increased by increasing the emission angle. That its intensity does not decrease further during the angle variation is related to the relative large uncertainty for small components near strong ones, here the ${\mathrm {Si}}_{\text {S}}$-component, the complex interplay of more than two components, and possible small contributions from Si-atom sites near the surface at this binding energy. The relative intensity of the ${\mathrm {Si}}_{\text {S}}$-component remains fairly constant upon variation of the surface sensitivity of the PES experiments indicating that the manifold of contributing Si-atom sites is neither dominated by the top-most Si atoms nor by Si bulk atoms. In contrast, the intensity of the Au silicide component increases monotonously with increasing surface sensitivity, i.e., the Si atoms bonded to Au are the top-most Si atoms. Thus, the observed nanowires, which are the top-most surface structures, are also not pure Si nanowires. This is further confirmed when comparing the intensity variation upon emission angle variation of the Au *4f* line and the most surface sensitive Si *2p* component, the ${\mathrm {Si}}_{\text {Au}}$-component (Fig. 6d). In general, the intensity of a line decreases when moving away from normal emission, which is also observed here. However, the intensity of the Au *4f* decreases slightly less than the one of the ${\mathrm {Si}}_{\text {Au}}$-component, so that the nanowire surface may be dominated by Au atoms bonded to Si atoms. Thus, it is evident that the here observed nanowires are formed by an Au–Si compound, i.e., they are Au silicide nanowires.

A rich manifold of different Au silicide structures have been reported due to the metastable nature of the Au silicides and the strongly varying preparation conditions, e.g., see reference^55^. For elongated Au silicide structures on Si(110), an orthorhombic and a hexagonal phase were already discussed based on the assumption that anisotropic strain leads to the growth of the elongated structures^29,31^. Both models would agree with the here observed growth of Au silicide nanowires, but no final conclusion on the exact atomic structure can be made based on the here obtained data and further investigations are needed for clarification, e.g., transmission electron microscopy studies may provide insight on the nanowire cross sections, which has proven useful for the structural analyses of similarly small nanowires of other silicides and of larger Au silicide nanoparticles^28,56^.

It has to be mentioned that for the above discussed PES data, no anisotropy of the ${\mathrm {Si}}_{\text {Au}}$-component had to be considered, although it may be expected due to the results fo the Au *4f* line. However, when one allows such an anisotropy during the fitting procedure, the same values regarding the areas of the components are obtained. This is illustrated in Fig. 6c, where circles correspond to fits using an anisotropic ${\mathrm {Si}}_{\text {Au}}$-component and lie directly on the corresponding crosses of the fits without anisotropy. Furthermore, the binding energy position of the Si bulk component is only marginally different upon Au silicide nanowire formation, shifted by nominally $0.02~{\mathrm {eV}}$. Thus, there is no change in band bending when moving from the clean reconstructed Si(110) surface to the Si(110) substrate covered by Au silicide nanowires.

#### Nanowire stability

**Figure 7:**
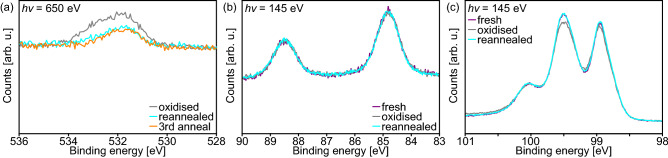
(**a**) O *1s* spectra of a more than $17~{\mathrm {h}}$ old Au silicide nanowires sample before and after reannealing. (**b**) Au *4f* and (**c**) Si *2p* spectra of a freshly prepared Au silicide nanowires sample, a slightly oxidised one, and a reannealed one. All spectra were obtained in NE and the spectra of a diagram were normalized to show the same intensity at low binding energies.

There are multiple overview spectra shown in Fig. 1c, which differ in their point of acquisition time with the sample stored in UHV between the measurements (about $5 \times 10^{-10}~{\mathrm {mbar}}$). The spectra are hardly discernible since they lay very well on top of each other. Even at the O 1*s* binding energy, there is only a very small increase of intensity more than $17~{\mathrm {h}}$ after the first measurement on the sample indicating a low reactivity of the Au silicide nanowires. Figure 7a shows a detailed measurement of the weak O 1*s* line of this oxidized state after more than $17~{\mathrm {h}}$. The area of the line can even be reduced by over $50\%$, when the sample is annealed for $30~{\mathrm {s}}$ at $600\,^\circ {\mathrm {C}}$ (reannealed in Fig. 7a). The remaining O is stable and cannot be significantly reduced by further annealing steps (Fig. 7a).

Taking a closer look at the corresponding Au *4f* and Si *2p* spectra shown in Fig. 7b,c, respectively, there are no changes in the Au *4f* spectra depending on the oxidation, but small variations observed for the Si *2p* spectra, i.a., of the intensity of the ${\mathrm {Si}}_{\text {Au}}$-component. Thus, the O did not react with Au atoms but partially with the Si atoms of the Au silicide nanowires. After the reannealing, the Si *2p* spectrum follows the one of the freshly prepared sample very well showing that all O could be desorbed from the Au silicide nanowires. The after the reanneal remaining O may have partially been on the sample directly after the preparation or may be adsorped in small Si oxide islands with very low surface coverage, so that it does only marginally influence the Si *2p* spectrum.

**Figure 8:**
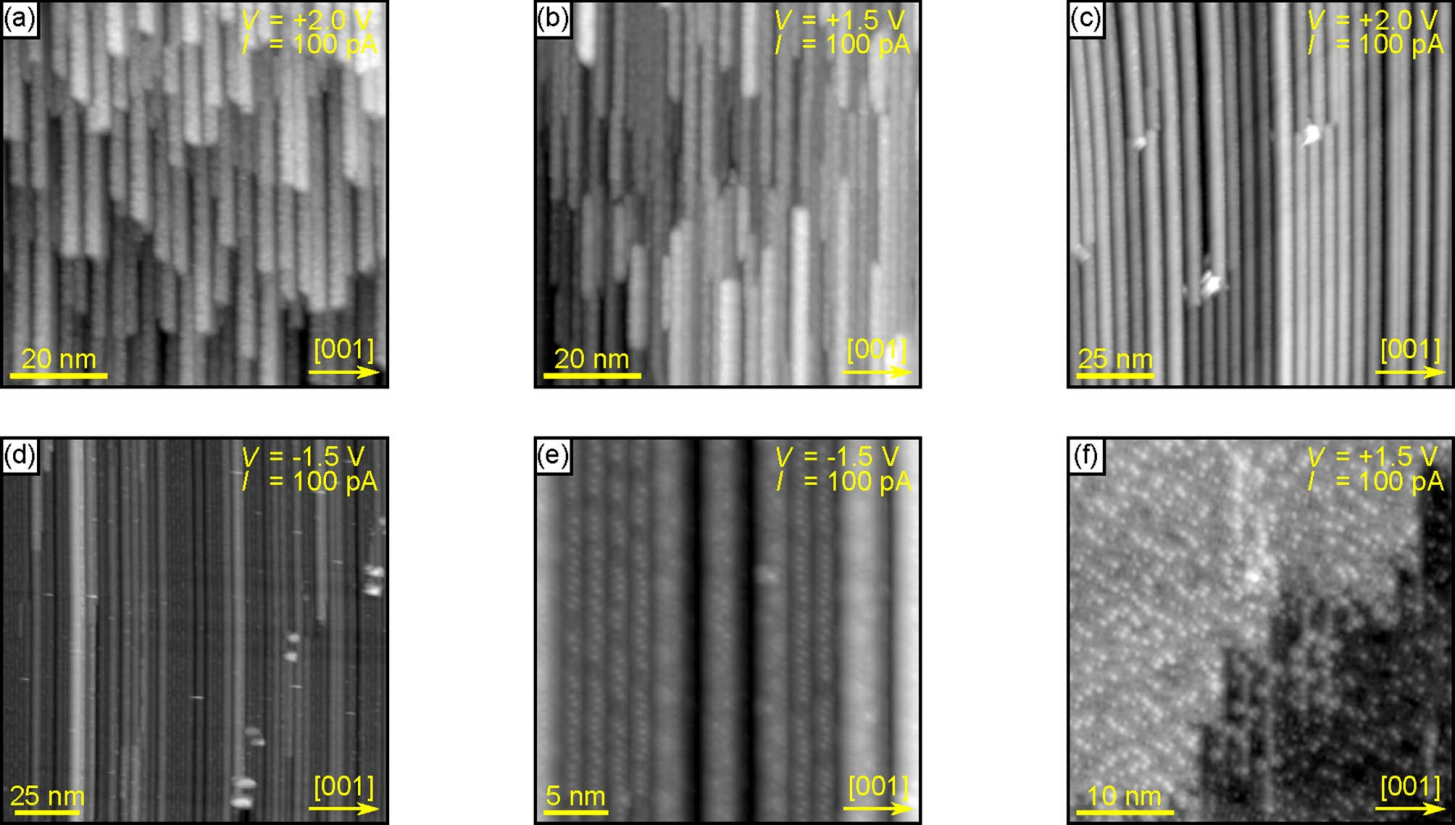
Chronologically measured STM images of the nanowire preparation shown in Fig. 3d after (**a**) 10 days in UHV, (**b**) an additional anneal at about $500\,^\circ {\mathrm {C}}$, (**c**) a subsequent anneal at about $750\,^\circ {\mathrm {C}}$ (13 days in UHV), and (**d**–**f**) reannealing at about $850\,^\circ {\mathrm {C}}$. The red line in (**e**) highlights triangular protrusions.

An even more long term stability was investigated using STM. As described in the introduction, bulk Au silicides are inherently unstable and their surfaces may decompose in the range of hours at room temperature^3^. In contrast, the here investigated Au silicide nanowires are still observed without any changes after 10 days (Fig. 8a). In agreement with the PES data, there are no changes of the nanowires for briefly annealing the nanowires below or to the preparation temperature (Fig. 8b). Additional annealing at higher temperatures up to $750\,^\circ {\mathrm {C}}$ lead to morphology changes consistent with the observations made in “Nanowire formation” section, i.e., nanowires with increased aspect ratios are found (Fig. 8c). Thus, the Au silicide nanowires are rather stable in UHV and up to $750\,^\circ {\mathrm {C}}$.

The situation drastically changes when Au silicide nanowire samples are annealed at $850\,^\circ {\mathrm {C}}$ (Fig. 8d–f). The density of the nanowires drastically decreases or they may completely vanish depending on the exact local conditions. The here observed surface structures are the $\left( 4 , 0\right) \times \left( {\overline{1}} , 3\right)$ superstructure in between the nanowires in Fig. 8d,e and the $1 \times 2$ reconstruction in Fig. 8f. Thus surface structures that form at lower Au coverages are observed indicating the agglomeration of Au into larger Au silicide or pure Au structures on the surface and/or the desorption of Au. This here observed destruction of the nanowire phase is in agreement with the vanishing of the corresponding streak structure in RHEED for temperatures above $800\,^\circ {\mathrm {C}}$ reported by Yamamoto^34^.

#### Comparison with other Au induced nanowires on Si(110)

The here observed Au silicide nanowires are not the first reported Au induced nanowires on Si(110). Thus, they have to be set in relation with the known structures.

Hong and co-workers reported the formation of nanowires for the deposition of $0.75~{\mathrm {ML}}$ Au and annealing for $30~{\mathrm {min}}$ at about $830\,^\circ {\mathrm {C}}$^42^. The nanowires formed in the $\left[ 1{\overline{1}}2\right]$ and $\left[ {\overline{1}}12\right]$ directions in areas up to $70 \times 90~{\mathrm {nm}}^2$ in contrast to the here observed single-domain growth of Au silicide nanowires over the whole sample. Furthermore, the appearance of the nanowires reported by Hong and co-workers is very similar to the one of the bare surface, so that very little or no Au is incorporated into them. This agrees very well with the here made observation of strong Au diffusion and possible Au desorption for annealing at $850\,^\circ {\mathrm {C}}$.

The workgroup of Dev reported nanowire formation for many multilayer thick Au films on B or H terminated Si(110) substrates after $30~{\mathrm {min}}$ annealing at the Au–Si eutectic temperature ($363\,^\circ {\mathrm {C}}$)^30,31^. Due to the much higher Au coverage and the long annealing, the nanowires are characterized by dimensions in the ${\upmu }{\mathrm {m}}$ range in contrast to the widths below $4~{\mathrm {nm}}$ and heights below $1~{\mathrm {nm}}$ found here for the Au silicide nanowires.

Very interestingly, the growth of pure Si nanowires was observed by Curiotto and co-workers for Au coverages larger than $1~{\mathrm {ML}}$ when the Si(110) substrate was heated to about $460\,^\circ {\mathrm {C}}$ and Au continuously deposited^50,51^. The nanowires formed by solid-liquid-solid epitaxy with gold islands as nucleation centres. Here, no indications of such nanowires or of Au islands were observed in the very local STM measurements and the globally averaging PES measurements. Thus, the studies by Curiotto and co-workers show the need for the here made in-depth structural analysis of the Au silicide nanowires to exclude pure Si nanowires, but another to be determined growth mechanism leads to the Au silicide nanowire formation.

## Conclusion

Nanowires formed by self-organization upon Au deposition on Si(110) substrates and annealing were investigated. They are made of an Au–Si compound and, by tuning the preparation conditions, their lengths are only limited by the substrate miscut implying huge aspect ratios due to their small widths of about 2 to $3~{\mathrm {nm}}$. The nanowires have a small but finite density of states at the Fermi level indicating metallicity but do not change the band bending behaviour at the surface compared to the clean reconstructed substrate. The Au silicide nanowires proved to be rather robust under UHV conditions and showed the possibility to be refreshed by reanneling. This low reactivity together with their single domain growth may be utilized to promote the growth of one-dimensionally ordered structures, e.g., of organic molecules. Such ordering was already shown for fullerenes on the clean substrate^57^, and may be possible for less inert molecules using the Au silicide nanowires as template. Furthermore, the electronic band structure of the nanowires may show one-dimensional characteristics due to the low heights and widths of the nanowires, which will have to be proven by angle-resolved PES. In general, the extreme dimensions of the nanowires set them apart from the other reported Au silicide nanostructures^28–32^, making them an intriguing system for further studies.

## Methods

The experiments were realised using two UHV chamber systems, the FlexPES Surface & Materials Science branch end station and the Scanning Probe Microscopy lab at MAX IV Laboratory. Both systems consists of preparation chambers equipped with LEED setups from OCI Vacuum Microengineering and dedicated analysis chambers. The sample preparation was the same in both UHV chamber systems.

The samples were cut from *n*-type Si(110) wafers. They were cleaned ex situ by rinsing with ethanol before transferring them into UHV. There, they were degassed for at least $2~{\mathrm {h}}$ at $600\,^\circ {\mathrm {C}}$. Then the samples were flash annealed up to $1150\,^\circ {\mathrm {C}}$ followed by slowly cooling them down from $800\,^\circ {\mathrm {C}}$ to get clean and well-ordered surfaces. The heating was realised by direct current through the samples and the temperature controlled by an infrared pyrometer with constant emissivity setting of $\varepsilon = 0.67$. Due to the often observed temperature gradients on the sample, the uncertainty of the temperature measurements should be estimated rather large ($\Delta T = 50\,^\circ {\mathrm {C}}$).

Gold was deposited on the samples by heating a W filament coated with Au. The deposited Au amount was determined by the deposition time and the deposition rate, which was calibrated by comparison to the known phase diagram of Au induced reconstructions on Si(110)^26,33,34^. The Au amount is given in ML with $1~{\mathrm {ML}}$ corresponding to the Si atom density of the unreconstructed Si(110) substrate ($\approx 9.59 \times 10^{-14}~{1/{\mathrm {cm}}^2}$) and equalling an Au film thickness of $\Theta \approx 0.161~{\mathrm {nm}}$. The samples were annealed after or during the Au deposition to enable the formation of ordered structures. The pressure remained below $1 \times 10^{-8}~{\mathrm {mbar}}$ during the whole preparation process.

The used VT AX STM from Scienta-Omicron was operated with W tips, which were in situ cleaned by electron bombardment. All images were obtained at room temperature in constant current mode using the sample voltages (*V*) and tunnelling currents (*I*) given in the STM images. The obtained images were carefully treated using mainly levelling filters, smoothing, and adjustment of the linear height scales using the Gwyddion software^58^. The $I\left( V\right)$-spectra were measured as point spectra with the tip situated above an Au silicide nanowire and without any intentional tip displacement after switching the feedback off.

The synchrotron photons for the PES measurements were delivered by the Surface & Material Science branch of the FlexPES beamline situated at the $1.5~{\mathrm {GeV}}$-ring of the MAX IV Laboratory. The photoelectrons were analysed using a Scienta SES-2002 spectrometer equipped with a two-dimensional MCP/CCD detector. The core-level spectra were analysed by least-square fitting using spin-orbit split (pseudo) Voigt profiles^59^. To reduce the number of fit parameters several constraints were used during the fitting procedure. For Si *2p* core-level spectra, the spin-orbit splitting and the intensity ratio of these components was fixed to $0.60~{\mathrm {eV}}$ and 2:1, respectively. Furthermore, only a constant and a Shirley-type background were considered. In contrast, an additional linear background had to be considered for reasonable fitting of the Au *4f* spectra. Due to simpler nature of the Au *4f* spectra without overlapping components, less restraints had to be used during the fitting procedure and, e.g., the determined spin-orbit splitting could be used for controlling the fit quality. As discussed in the main part of this report, deviations from the expected 4:3 intensity ratio of the spin-orbit split components had to be considered. Binding energy scales were calibrated by measuring the Fermi edge on the polycrystalline Ta clamps of the sample holder also covered slightly by Au.
